# Manufacturing ZrB_2_–SiC–TaC Composite: Potential Application for Aircraft Wing Assessed by Frequency Analysis through Finite Element Model

**DOI:** 10.3390/ma13102213

**Published:** 2020-05-12

**Authors:** Behzad Mohammadzadeh, Sunghoon Jung, Tae Hyung Lee, Quyet Van Le, Joo Hwan Cha, Ho Won Jang, Sea-Hoon Lee, Junsuk Kang, Mohammadreza Shokouhimehr

**Affiliations:** 1 Department of Landscape Architecture and Rural Systems Engineering, Seoul National University, Seoul 08826, Korea; Behzad3049@snu.ac.kr; 2 Research Institute of Agriculture and Life Sciences, Seoul National University, Seoul 08826, Korea; 3 Department of Materials Science and Engineering, Research Institute of Advanced Materials, Seoul National University, Seoul 08826, Korea; gns84@snu.ac.kr (S.J.); sunshinety@snu.ac.kr (T.H.L.); hwjang@snu.ac.kr (H.W.J.); 4 Advanced Nano Surface Department, Surface Technology Division, Korea Institute of Materials Science, Changwon 51508, Korea; 5 Institute of Research and Development, Duy Tan University, Da Nang 550000, Vietnam; 6 Innovative Enterprise Cooperation Center, Korea Institute of Science & Technology, Hwarangro 14-gil, Seongbuk-gu, Seoul 02792, Korea; jhcha@kist.re.kr; 7 Division of Powder/Ceramics Research, Korea Institute of Materials Science, Changwon 51508, Korea

**Keywords:** ultra-high temperature ceramic, spark plasma sintering, nano-indentation, composite, vibration analysis, FEM analysis

## Abstract

This study presents a new ultra-high temperature composite fabricated by using zirconium diboride (ZrB_2_), silicon carbide (SiC), and tantalum carbide (TaC) with the volume ratios of 70%, 20%, and 10%, respectively. To attain this novel composite, an advanced processing technique of spark plasma sintering (SPS) was applied to produce ZrB_2_–SiC–TaC. The SPS manufacturing process was achieved under pressure of 30 MPa, at 2000 °C for 5 min. The micro/nanostructure and mechanical characteristics of the composite were clarified using X-ray diffraction (XRD), field-emission scanning electron microscopy (FESEM), and nano-indentation. For further investigations of the product and its characteristics, X-ray fluorescence (XRF) analysis and X-ray photoelectron spectroscopy (XPS) were undertaken, and the main constituting components were provided. The composite was densified to obtain a fully-dense ternary; the oxide pollutions were wiped out. The mean values of 23,356; 403.5 GPa; and 3100 °C were obtained for the rigidity, elastic modulus, and thermal resistance of the ZrB_2_–SiC–TaC interface, respectively. To explore the practical application of the composite, the natural frequency of an aircraft wing considering three cases of materials: (i) with a leading edge made of ZrB_2_–SiC–TaC; (ii) the whole wing made of ZrB_2_–SiC–TaC; and (iii) the whole wing made of aluminum 2024-T3 were investigated employing a numerical finite element model (FEM) tool ABAQUS and compared with that of a wing of traditional materials. The precision of the method was verified by performing static analysis to obtain the responses of the wing including total deformation, equivalent stress, and strain. A comparison study of the results of this study and published literature clarified the validity of the FEM analysis of the current research. The composite produced in this study significantly can improve the vibrational responses and structural behavior of the aircraft’s wings.

## 1. Introduction

Ultra-high temperature ceramics (UHTCs) are refractory materials presenting outstanding mechanical properties at elevated temperatures beyond 2000 °C [1,2,3]. They have been used for possible thermal protection systems, coating of objects exposed to high temperatures, and biomedical applications [4,5,6,7]. A few natural elements exist having ultra-high melting points over 3000 °C such as carbon, rhenium, osmium, tungsten, and tantalum [8,9,10]. Also, limited chemical compounds having high-temperature resistance have been achieved and utilized for various applications, e.g., thorium dioxide (ThO_2_), tantalum diboride (TaB_2_), zirconium diboride (ZrB_2_), tantalum carbide (TaC), hafnium carbide (HfC), zirconium carbide (ZrC), titanium carbide (TiC), etc. [11,12,13,14].

The huge demand for high-temperature resistant materials for industries, especially aerospace, has motivated the Air Force Materials Laboratory to develop a new class of materials tolerating the harsh environment of proposed hypersonic vehicles such as Dyna-soar and the Space Shuttle at Man Labs Incorporated [15]. Assessment of the refractory properties of binary ceramics showed that the metal borides, carbides, and nitrides possessed the significant thermal conductivity and oxidation resistance as well as appropriate mechanical strength in the case of using small grain size. Among the aforementioned materials, ZrB_2_ and HfB_2_ in composites comprising ~20% volume SiC have presented the most efficient and practical applications. A significant number of research studies reported in the literature dealt with improving the performance of ZrB_2_ and HfB_2_ despite having numerous works that have been undertaken to characterize the nitrides, oxides, carbides, etc. [16,17,18,19,20]. There are several documented studies presenting the diborides utilization for high thermal conductivity and low melting points. These unique properties of such ceramics provide significant thermal resistance making them ideal candidates for elevated-thermal applications [21,22,23]. Among the UHTCs, the Zr-based diborides have been developed with the aim of tolerating ambient loads and elevated temperatures which can be experienced by leading vehicle edges in sustained hypersonic flight [24,25]. The surfaces of aircraft vehicles are exposed to drastically elevated temperatures and high flow-rate oxidation. It has been documented that ZrB_2_-based UHTCs can retain shape under high-temperature surroundings because of their significant oxidation resistance and high melting point [26,27,28].

ZrB_2_ has been considered one of the most promising thermal protection materials and employed for a wide range of structural and industrial applications [29,30,31]. From the results given in the literature, it has been figured out that ZrB_2_-based UHTCs possess an outstanding oxidation resistance, rigidity, and fracture toughness. It was observed that the oxidation resistance of ZrB_2_–SiC composite was supreme in the air up to 1500 °C boosting the strength by healing the surface flaws [32,33]. The significant oxidation resistance of ZrB_2_–SiC beyond 1800 °C has been also reported presenting outstanding integrity of the underlying microstructure under specific situations [34,35]. Furthermore, huge efforts have been assigned to the assessment of high-temperature mechanical properties of ZrB_2_-based ceramics, particularly by incorporating various additives. In this regard, several studies have been reported on the investigation of the flexure strength of ZrB_2_-based ceramics at high temperatures by employing the three-point bending test. The results showed that the flexure strength, stiffness, thermal, and corrosion resistance were relatively decreased with increasing the temperature [36,37,38]. Among various additives included to UHTCs for improving their properties, TaC is unique because of its high melting point (~3880 °C) which is among the highest for binary compounds. Furthermore, TaC has a microhardness of 1600–2000 kg/mm^2^, and an elastic modulus of 285 GPa.

This study focuses on the production of a new type of UHTCs having superior characteristics to the existing conventional counterparts and newly manufactured ZrB_2_-based materials [39,40,41,42,43,44,45,46,47,48,49]. Based on the investigations through the characteristics of different additives, this study employed TaC nanopowder to enhance the mechanical properties of ZrB_2_–SiC for industrial and structural applications. Thereafter, to visualize the practical application of the presented manufactured composite, it was adopted for the aerospace applications. For this aim, the finite element (FE) method which has been frequently used as an outstanding tool for a wide range of applications such as modeling materials, and structural and mechanical components, vibration analysis, buckling analysis, crack propagation, and blast analysis of structures and materials, was taken into account [50,51,52,53,54,55,56,57,58,59,60]. In this regard, the FE package ABAQUS was adopted to evaluate the natural frequencies of an aircraft wing of aluminum 2024-T3 and the leading edge made of the ZrB_2_–SiC–TaC.

## 2. Experimental Setup

### 2.1. The Manufacturing Process of ZrB_2_–SiC–TaC

To manufacture the ZrB_2_–SiC–TaC composite, ZrB_2_, SiC, and TaC were mixed in the form of powder with the volume fractions of 70%, 20%, and 10%, respectively. The purity of ZrB_2_ was greater than 99.8% with the particle size smaller than 2 μm. SiC had a particle size of <3 μm with a purity of 99.2%, while the TaC powder size was smaller than 100 nm having a purity of 99.5%. The mixing of the starting materials was carried out inside an ultrasonic bath with ethanol lasting for 80 min. The admixture was completely dried using a magnetic hot plate and a stove. When the mixture was thoroughly loaded in the mold, the sintering process was carried out by spark plasma sintering (SPS) subjected to 2000 °C under 30 MPa pressure for 5 min. The obtained pallet with a thickness of 6 mm and a diameter of 2.4 mm was unfolded by grinding using a diamond grinding disk and eliminating its graphite foil cover.

### 2.2. Analysis and Characterization

To characterize the crystal structure of the prepared composite, a high-resolution X-ray diffractometer (XRD) was performed on a Bruker D8 Advance instrument (Bruker, Billerica, MA, USA). To obtain the mechanical characteristics of the composite the instrumented nano-indentation test was adopted by employing the Berkovich indenter. ZrB_2_–SiC–TaC was investigated by the Oliver-Pharr method to reveal its mechanical and thermal properties such as modulus of elasticity (E), Poisson’s ratio (ν), rigidity, and fracture toughness, and the load-displacement relationship. For this aim, XRD, and nano-indentation were utilized. Field-emission scanning electron microscopy (FESEM) images were attained using a SUPRA 55VP and Sigma Carl Zeiss (Zeiss, Oberkochen, Germany) instrument equipped with energy-dispersive X-ray spectroscopy (EDS). X-ray photoelectron spectroscopy (XPS) was carried out using an Al Kα source (Sigma probe, VG Scientifics). The field emission-electron probe microanalyzer (FE-EPMA) was acquired using a JXA-8530F, JEOL (Tokyo, Japan). X-ray fluorescence (XRF) spectroscopy was achieved by an XRF-1800, Shimadzu (Kyoto, Japan).

## 3. Potential Applications and Computational Model

**Figure 1:**
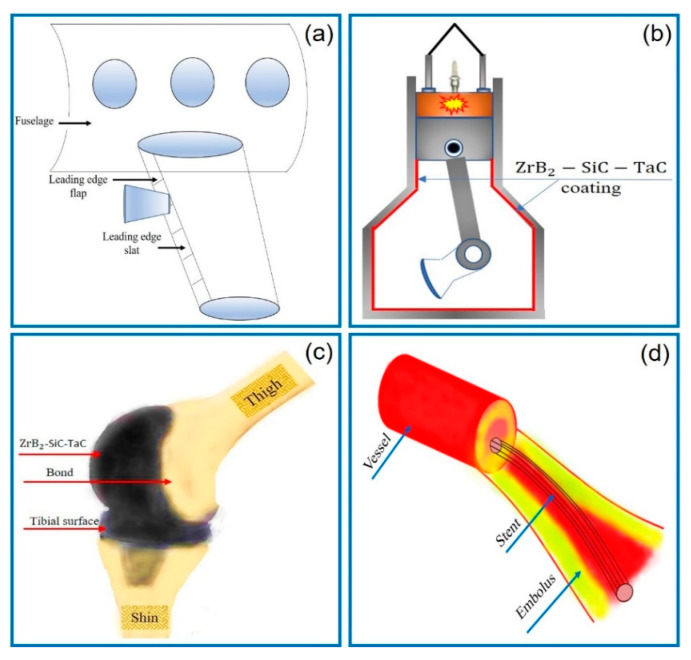
Potential applications of ZrB_2_–SiC–TaC for (**a**) aerospace, (**b**) combustion cylinder of the car engine, (**c**) artificial knee joint, and (**d**) stent.

The prominent requirements of mechanical, thermal, and corrosion properties for the materials used for aerospace applications limits the choice of materials to carbides, borides, nitrides of the transition metals, etc. Among the available materials, TaC is unique because of its high melting point over 3000 °C. Moreover, the TaC-based composites provide enhanced mechanical characteristics compared with other UHTCs. The composite of this study, ZrB_2_–SiC–TaC, can be an appropriate alternative for current commercial materials for long-term aerospace applications. The components of the leading edge and nose cap of aircraft are exposed to temperatures higher than 2000 °C and subjected to complex stresses. The illustration of the possible applications of the materials of this research in aircraft wings is provided in Figure 1a.

Furthermore, the refractory property and high toughness of ZrB_2_–SiC–TaC enable it to be used for car engines and brakes, nuclear reactors, and coating on wooden structures. It can also be used for electric and electronic devices because ZrB_2_ acts as an electrical conductor, while SiC functions as an electrical ceramic. Figure 1b illustrates the potential application of this ceramic for the car industry used as an internal coating of the combustion chamber. Due to the high fracture toughness, corrosion, and oxidation resistance of the presented ceramic, it can also be utilized for biomedical applications, e.g., knee joints (Figure 1c). Furthermore, it can be applied for stent fabrication to keep the arteries passageway open. Figure 1d shows the possible biomedical applications for the ZrB_2_–SiC–TaC in artificial knee replacement and stent used to open clogged arteries.

To examine the practical applications of ZrB_2_–SiC–TaC, this study adopted the commercially available finite element package ABAQUS to investigate the natural frequencies of the aircraft wing with a leading edge made of the ceramic. The solid elements were adopted for the geometrical modeling of the wing. There are two models in terms of the materials: i) the case that the aluminum 2024-T3 was specified to the global body of the wing while the composite of this study was allocated to the leading-edge; and ii) the wing made totally with aluminum 2024-T3. It is worth noting that in the case of having a wing made of both materials, the volume fraction of the ZrB_2_–SiC–TaC used for leading-edge is ~18%, and for aluminum used in the rest of the wing is ~72%. The mechanical properties of the materials used for the wing are provided in Table 1.

**Figure 2:**
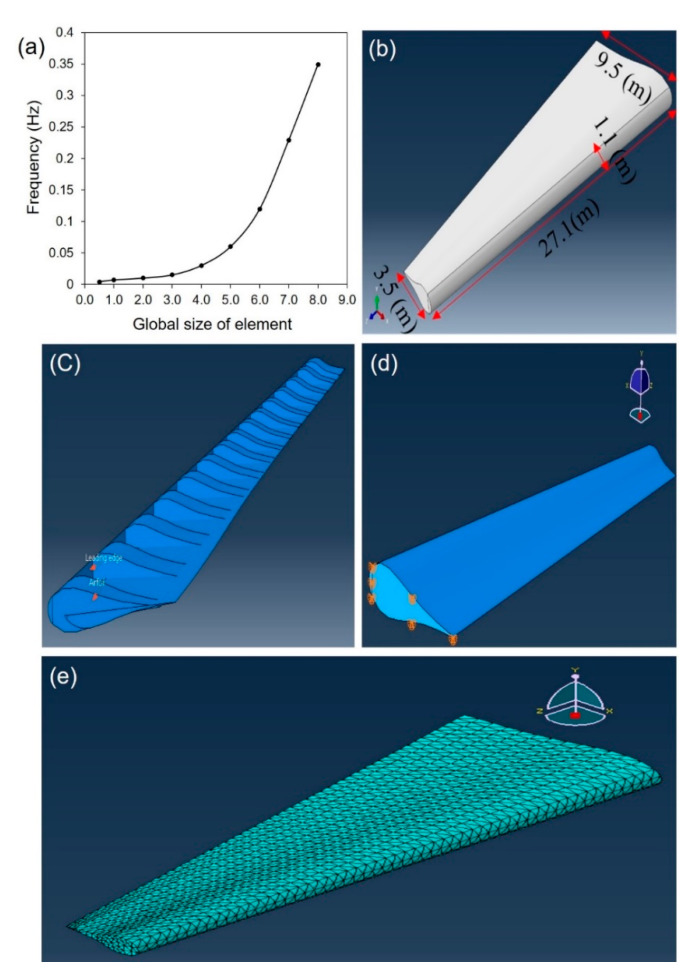
The mesh size evaluation and model of the aircraft wing in ABAQUS: (**a**) convergence study evaluating the appropriate mesh size for the wing, (**b**) wing dimensions, (**c**) internal view of wing showing the inside construction, and (**d**) clamped boundary conditions applied on the wing end, (**e**) meshed model.

There are two commonly used elements for meshing 3D models: the brick element and the tetrahedron element. It has been documented that brick elements can show better performance compared with the tetrahedron. However, for the cases of complex geometries, the probability of geometry degradation exists when meshing the model with brick elements. In addition, the number of nodes of brick elements is higher than the number of elements which significantly increases the computational time. Re-meshing of the brick elements is difficult and hence may not be suitable for large deformation problems. Therefore, the tetrahedron element, the 10-node quadratic tetrahedron (C3D10), was adopted for the numerical model of this study. To find the appropriate mesh size, the convergence study was performed and the corresponding graph is illustrated in Figure 2a.

It can be seen from Figure 2a that as the size of elements increases the convergence of the results decreases such that after the size of 3.0, the rate of divergence exponentially increases. In this study, to achieve reliable results, the global size of 0.5 was adopted for the mesh size. The geometry of the wing model in ABAQUS is illustrated in Figure 2b while the open-view of the wing showing the airfoils inside the wing is provided in Figure 2c. The boundary conditions shown on the wing end which are connected to aircraft fuselage is provided in Figure 2d, and the meshed model is given in Figure 2e.

## 4. Results

### 4.1. Characterization of ZrB_2_–SiC–TaC

**Figure 3:**
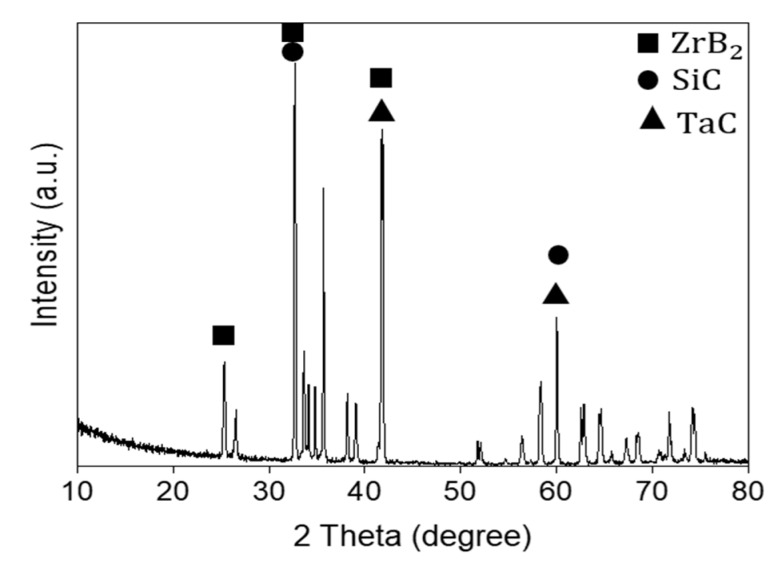
X-ray diffraction (XRD) pattern of ZrB_2_–SiC–TaC.

To provide a better insight into the crystal structure and determine the characteristics of ZrB_2_–SiC–TaC, several methods were employed, namely nano-indentation, XRD, XRF, FE-EPMA, and FESEM. The XRD pattern of the prepared ceramic is shown in Figure 3.

**Figure 4:**
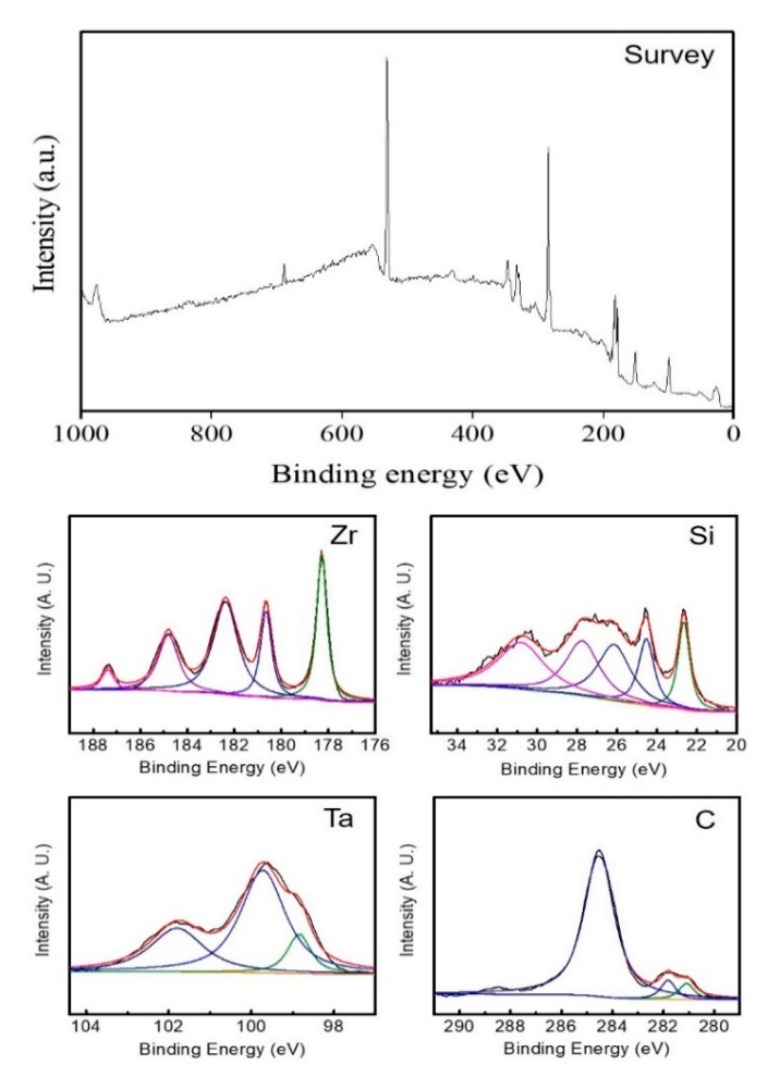
X-ray photoelectron spectroscopy (XPS) of ZrB_2_–SiC–TaC and its constituent compounds.

XPS analysis of the elements existing in ceramic and the composition of ZrB_2_–SiC–TaC is illustrated in Figure 4.

**Figure 5:**
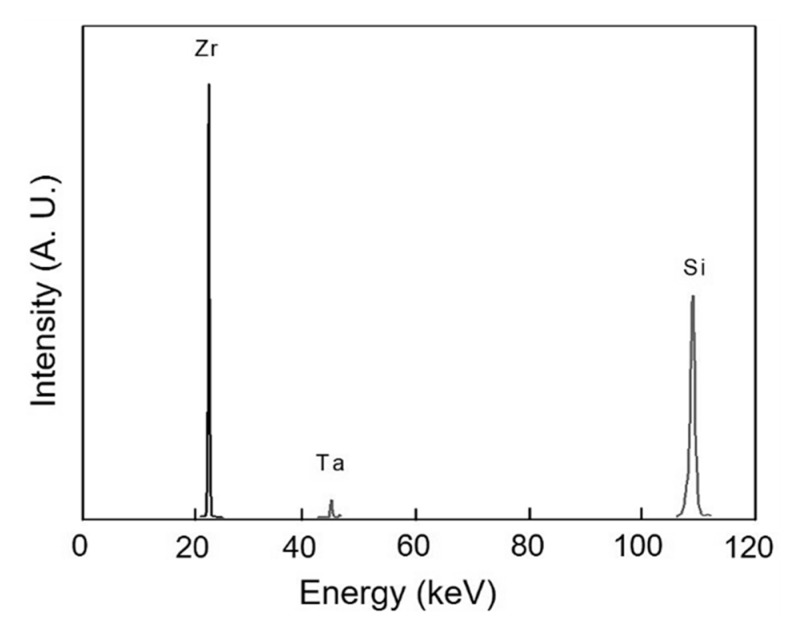
X-ray fluorescence (XRF) spectroscopy of ZrB_2_–SiC–TaC.

**Figure 6:**
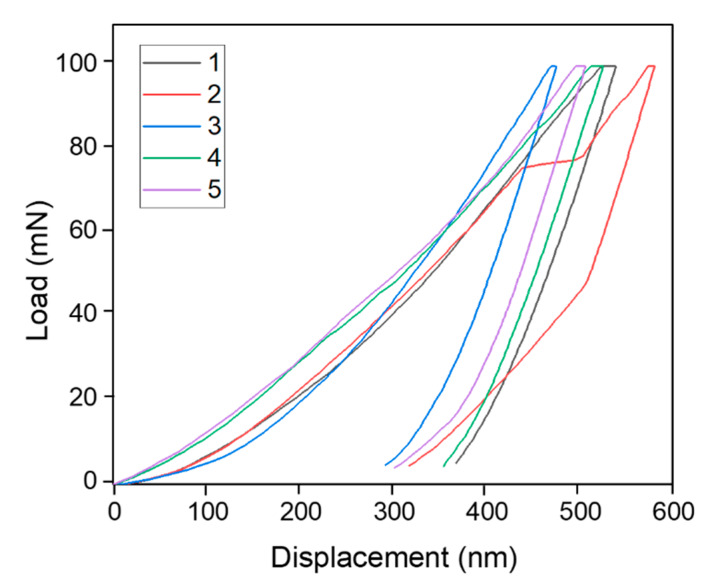
Nano-indentation of ZrB_2_–SiC–TaC.

The graph showing the XRF of the composite of this study is given in Figure 5 while the nano-indentation of ZrB_2_–SiC–TaC showing the load-displacement relationship of this composite is provided in Figure 6.

Furthermore, the non-destructive analytical technique of XRF was used to determine the elemental composition of ZrB_2_–SiC–TaC. The characteristics of the ZrB_2_–SiC–TaC are provided in Table 2.

**Figure 7:**
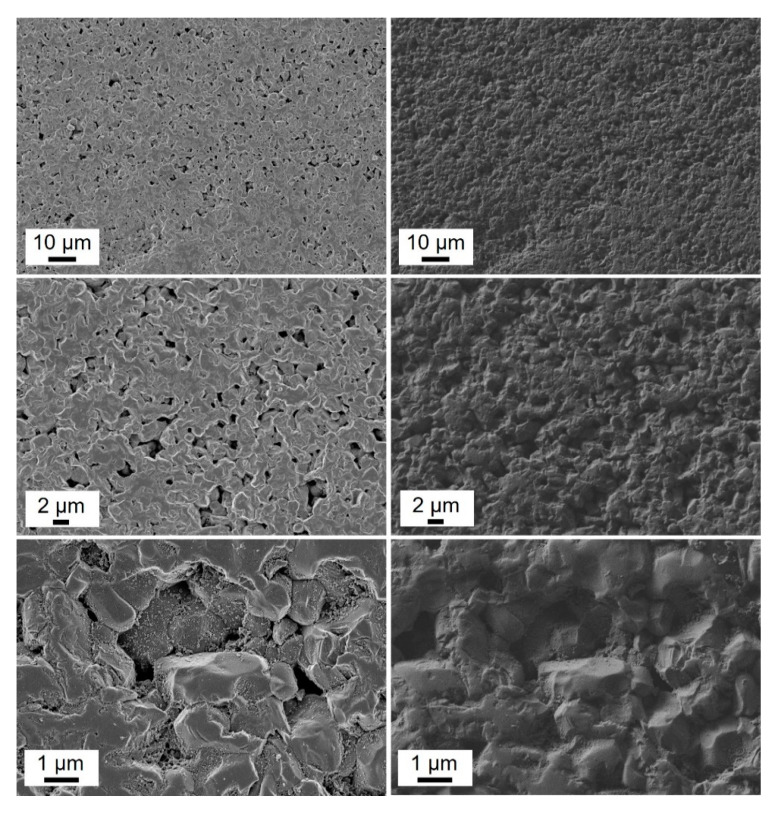
Different magnification of field-emission scanning electron microscopy (FESEM, **left**), and corresponding secondary electron detector (**right**) images of ZrB_2_–SiC–TaC.

Table 2 shows that ZrB_2_–SiC–TaC has very high rigidity, elastic modulus, and thermal resistance. These outstanding properties together with its high thermal and oxidation resistance expands its utilization for industrial and structural applications. Figure 7 shows the FESEM images of ZrB_2_–SiC–TaC composite.

**Figure 8:**
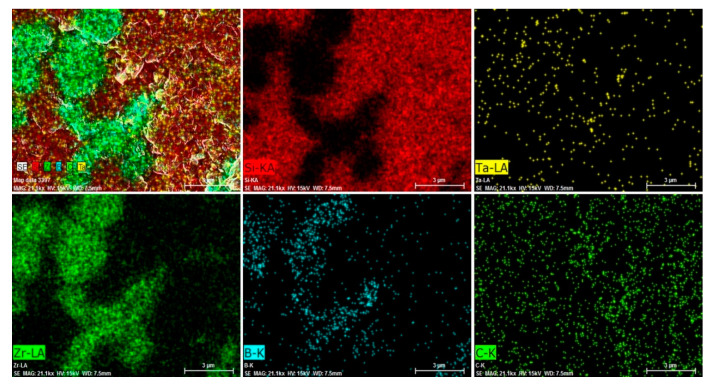
Energy-dispersive X-ray spectroscopy (EDS) map of ZrB_2_–SiC–TaC.

The EDS elemental map of the composite presenting zirconium, boron, carbon, silicon, and tantalum is provided in Figure 8.

**Figure 9:**
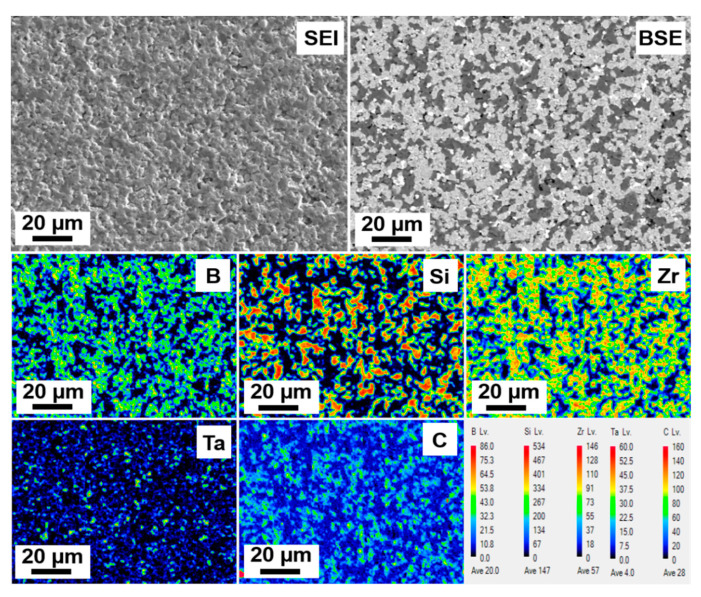
Field-emission-electron probe microanalysis (FE-EPMA) of ZrB_2_–SiC–TaC and its constituent compounds.

FE-EPMA was employed to explain and illustrate the carboniferous dopant existence in terms of primary graphite as well as recently-created zirconium diboride composite after sintering procedure (Figure 9).

### 4.2. Computational Results

To evaluate the applicability of the material of this study, we applied it to the leading edge of the aircraft wing and total body of the wing. The aim was to investigate the natural frequency of the wing by employing the finite element model (FEM) method through ABAQUS.

#### 4.2.1. Method Validity

To evaluate the validity of the computational method of this study, the results given in the literature [50] dealing with the analysis of an aircraft wing was adopted to be compared with the current study. The example problem given in the aforementioned reference was modeled in this study. The Aluminum 2024-T3 was considered for the material of the wing and static analysis was performed to obtain the total deformation, equivalent stress, and equivalent strain. For the static analysis, the pressure force of 500 Pa was applied to the bottom surface of the wing at the center of the pressure. One end of the wing was completely constrained for all the degrees of freedom as it is embedded inside the fuselage while the other end was left free with six degrees of freedom. Thereafter, the results were compared with those of literature as provided in Table 3.

The last row of Table 3 shows the variation of the results of the current study and literature. The small amount of variation shows that the numerical model of this study could provide an acceptable prediction of the results and was in good agreement with literature. Therefore, the validity of the FEM analysis of the current study employing ABAQUS was approved. It can be adopted for further analysis to evaluate the natural frequencies of the aircraft wing considering the material produced in this study, ZrB_2_–SiC–TaC.

#### 4.2.2. The Natural Frequency of the Wing

**Figure 10:**
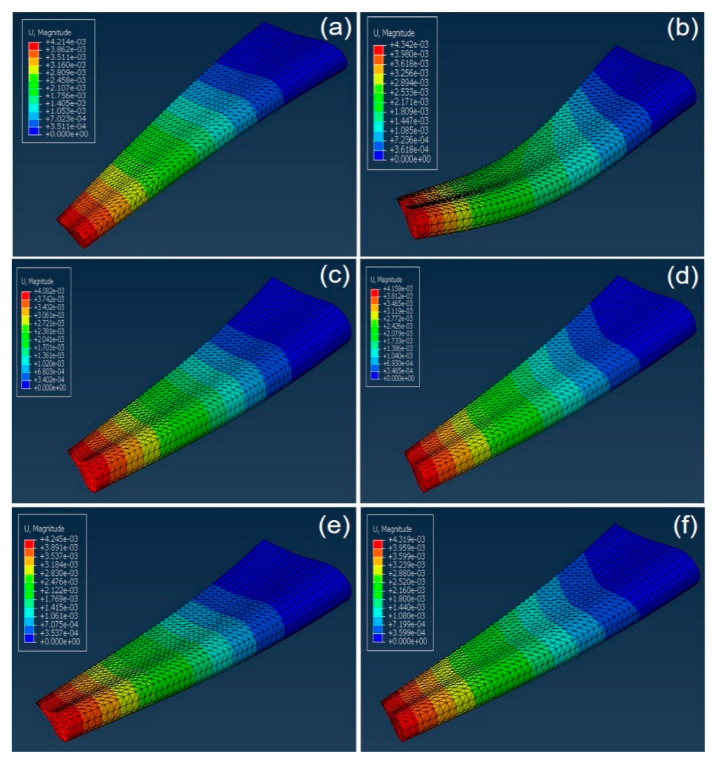
First two modes of wing natural frequency of (**a**) ZrB_2_–SiC–TaC for the leading edge (model I), (**b**) ZrB_2_–SiC–TaC for the leading edge (model II), (**c**) ZrB_2_–SiC–TaC for the body of the wing (model I), (**d**) ZrB_2_–SiC–TaC for the body of wing (model II), (**e**) aluminum for the body of wing (model I), and (**f**) aluminum for the body of wing (model II).

The illustrations of the first two modes of natural frequencies of wings are provided in Figure 10 for three cases: (i) ZrB_2_–SiC–TaC as the leading edge and aluminum for the rest of the wing-body, (ii) ZrB_2_–SiC–TaC used for all the body of wing, and (iii) aluminum used for the wing. As can be seen from Figure 10, the maximum displacement was observed at the free end of the wing. Similar mode shapes were obtained for the wings made of pure aluminum and ZrB_2_–SiC–TaC.

## 5. Discussions

### 5.1. Experiments

Performing a set of experimental works resulted in the production of a refractory material called ZrB_2_–SiC–TaC to provide superior thermal and mechanical properties for potential applications in structural, mechanical, aerospace, and biomedical areas. Thereafter, the composite was characterized by employing nano-indentation, XRD, XRF, and XPS. The densification of the material was completely achieved through the SPS process. Only peaks of zirconium and boron could be obtained allocated to the matrix of ZrB_2_. The peaks of silicon and carbon could be only detected as SiC additive. Figure 3 provides XRD of the ZrB_2_–SiC–TaC. Small values of the sedentary shaped ZrB_2_ phase enhanced the assumption indicating the bounded reaction between TaC and the face contamination of zirconium diboride–silicon carbide. Results revealed that the intensity of the TaC top point in the sintered compound was smaller than the powder admixture. The relative density of ZrB_2_–SiC–TaC was 98.5% through the SPS process under the temperature of 2000 °C and 30 MPa pressure for 5 min. Despite achieving the theoretical density for the composite of this study, the remaining 1.5% porosity in the as-sintered experimental sample could be attributed to insufficient sintering temperature or the absence of any metallic, carbonaceous, or other types of sintering aids. However, because of the negative effects of employing the sinter additives or higher sintering temperatures on the mechanical performance of sintered parts, a value of 98.5% would be admissible.

The XPS images of the composite and constituent compounds are provided in Figure 4. Having the XPS analysis of the materials, as a very advanced and accurate analysis tool, it was found that the purity of the composite is excellent, and no other compound was produced. Similar results were obtained by XRF analysis (Figure 5). Among the constituent materials, the highest intensity belonged to ZrB_2_ corresponding to the binding energy of approximately 178 (eV). The lowest intensity attributed to TaC with a magnitude of 2300 which occurred on the binding energy equal to 23 (eV). 

It has been documented that there is a possibility of the creation of new compounds as well as the desired main composite during the manufacturing process. For example, Ghassemi Kakroudi and co-workers [38] detected two other compounds including ZrC and TaSi_2_ during the hot process manufacturing of ZrB_2_–SiC–TaC. They explained that it could be because of the formation of oxide impurity on the surface of ZrB_2_ powders as the main component of the mixture. They suggested the chemical reaction formula given in Equation (1) for the verification of this outcome [38]:

2SiC + TaC + ZrO_2_ → TaSi_2_ + ZrC + 2CO (1)

However, from the outcomes obtained from XRD, XPS analysis of the current study, no extra in situ formation of new compounds was observed except the main composite of desire. This superior result was achieved due to employing the advanced SPS manufacturing process which is preferable to the other existent manufacturing processes.

Figure 6 shows the load-displacement relation of the material obtained from the nano-indentation technique. As can be seen, the result was achieved through five loading-unloading processes. After each cycle, the amount of displacement increased with respect to the same loading amounts which shows the slight reduction in the rigidity of the material. By using the nano-indentation graphs, the characteristics of the composite of this study were attained. The mechanical and thermal characteristics of the composite and ZrB_2_–SiC–TaC interface are given in Table 2 shows a substantial reduction in the minimum and mean rigidity and modulus of elasticity of the interface with respect to those of zirconium diboride, silicon carbide, and tantalum carbide phases. It was observed that the existence of residual porosities and oxides could result in a decrease in rigidity and modulus of elasticity at the interface. It was found that SiC dominated in the regarding indent because of the indentation head position. For example, the case can be considered as the principal segment of the projected region positioned within silicon carbide for which the force-displacement relation tended to that of SiC. By contrast, when the indentation was positioned within the ZrB_2_ phase, the properties of the force-displacement relationship tended to ZrB_2_. The same pattern was also observed for the TaC for which the material characteristics presented those of TaC when the indentation placed within TaC.

FESEM images of the surface of the composite are shown in Figure 7 comprising grey, dark, and bright areas correspondence to ZrB_2_, SiC, and TaC grains, respectively. There is a good consistency with the volume ratios of each phase in the powder mixture. Besides, this microstructural result was in good agreement with the relative density value of 98.5% for the sintered bulk. EDS of the composite also confirms the integrity and presence of the constituent elements in the ZrB_2_–SiC–TaC (Figure 8). The FE-EPMA map also shows the areal information on the properties of the composite and its chemical components provided in Figure 9. These results demonstrated the distribution map of each component and the whole composition of ZrB_2_–SiC–TaC. 

### 5.2. Finite Element Method

The numerical analysis of the aircraft wing was performed to investigate the effect of the application of ZrB_2_–SiC–TaC on the natural frequency of the aircraft wing. The first two shape modes of the wings corresponding to different cases from the viewpoint of the material are provided in Figure 10. As can be seen from Figure 10, the free end of the wing sustained the highest displacement. To provide a better understanding of the efficiency of the material of this study, the amounts of natural frequencies corresponding to the first three mode shapes, the most important modes, are provided and compared in Table 4.

According to the results given in Table 4, the natural frequency of the aircraft wing is changed by using ZrB_2_–SiC–TaC. It was observed that employing the material of this study for the leading edge of the wing increased the natural frequency by over 50%. Moreover, it was seen that having used the presented composite instead of aluminum for the wing resulted in a small increase in natural frequencies. Using the composite of this study significantly enhances the stiffness of the wing as well as its thermal resistance.

## 6. Conclusions

This presented a new type of ultra-high temperature ceramic, ZrB_2_–SiC–TaC, having the thermal resistance beyond 3000 °C, and superior mechanical properties. The purpose of the study was to produce new material for structural and aerospace applications. The designed composite was produced by combining zirconium diboride, silicon carbide, and tantalum carbide sintered by spark plasma sintering. This new composite was characterized through the nano-indentation, X-ray diffractometer, X-ray fluorescence spectrometer, field emission-electron probe microanalyzer, and field-emission scanning electron microscopy. This composite possesses a melting point beyond 3000 °C, elastic modulus, and rigidity. Its possible application in aerospace was illustrated. To depict the workability and applicability of the material of this study, the computational method was adopted to model the aircraft wing through finite element model analysis employing ABAQUS. In this regard, three cases were considered from the viewpoint of specifying ZrB_2_–SiC–TaC in the wing: i) only applied to the leading edge of the wing, ii) considered for the wing as a whole, and iii) only aluminum 2024-T3 applied to the wing without consideration of ZrB_2_–SiC–TaC. Comparison studies between the results showed that employing ZrB_2_–SiC–TaC enhances the structural performance of the wing and changes its natural frequency substantially when it is considered as a leading edge of the wing. When ZrB_2_–SiC–TaC was applied to the wing as a whole, a small variation was observed with the case of wing fully made of aluminum 2024-T3. It can be concluded that the best case is to apply this composite for the leading edge of the wing. 

## Figures and Tables

**Table 1:** Rigidity and elastic modulus of each phase.

Mechanical Property	Material	Value
Elastic modulus (GPa)	ZrB_2_–SiC–TaC	403.5
	Aluminum 2024-T3	73.1
Poisson’s ratio	ZrB_2_–SiC–TaC	0.29
	Aluminum 2024-T3	0.33
Density (kg/m^3^)	ZrB_2_–SiC–TaC	3000
	Aluminum 2024-T3	2780

**Table 2:** Mechanical and thermal properties of the material interface.

Mechanical Property	Phase	Min	Max	Mean
Rigidity (GPa)	ZrB_2_–SiC–TaC Interface	16218	26125	23356
Elastic modulus (GPa)	ZrB_2_–SiC–TaC Interface	359.4	460.1	403.5
Thermal resistance (°C)	ZrB_2_–SiC–TaC Interface	3000	3200	3100

**Table 3:** Comparison between the results of static analysis of aircraft wing obtained from the current study and literature [50].

Entry	Total Deformation (mm)	Equivalent Stress (MPa)	Equivalent Strain
Reference [50]	6.7377	16.034	0.00023
Current	6.8893	16.488	0.00075
Variation%	2.25	2.75	2.26

**Table 4:** First three modes corresponding to the aircraft wing natural frequencies.

Material	Mode I (Cycle/Time)	Mode II (Cycle/Time)	Mode III (Cycle/Time)
ZrB_2_–SiC–Tac	2.67 × 10^−3^	1.13 × 10^−2^	1.14 × 10^−2^
Al	2.56 × 10^−3^	9.91 × 10^−3^	1.08 × 10^−2^
ZrB_2_–SiC–Tac + Al	5.79 × 10^−3^	2.24 × 10^−2^	2.44 × 10^−2^
Variation% (Al/Zr)	4.12	12.30	5.26
Variation% (Al/Zr–Al)	55.79	55.76	55.74
